# General intelligence is associated with subclinical inflammation in Nepalese children: A population-based plasma proteomics study

**DOI:** 10.1016/j.bbi.2016.03.023

**Published:** 2016-08

**Authors:** Sun Eun Lee, Keith P. West, Robert N. Cole, Kerry J. Schulze, Lee Shu-Fune Wu, James D. Yager, John Groopman, Parul Christian

**Affiliations:** a Center for Human Nutrition, Department of International Health, Johns Hopkins Bloomberg School of Public Health, 615 N. Wolfe Street, Baltimore, MD 21205, USA; b Mass Spectrometry and Proteomics Facility, Department of Biological Chemistry, Johns Hopkins School of Medicine, 733 N. Broadway Street, Baltimore, MD 21205, USA; c Department of Environmental Health Sciences, Johns Hopkins Bloomberg School of Public Health, 615 N. Wolfe Street, Baltimore, MD 21205, USA

**Keywords:** Intelligence, Universal Nonverbal Intelligence Test, Plasma proteomics, Mass spectrometry, Inflammation, Children, Nepal, Cohort study

## Abstract

Improving child cognition in impoverished countries is a public health priority. Yet, biological pathways and associated biomarkers of impaired cognition remain poorly understood and largely unknown, respectively. This study aimed to explore and quantify associations between functional plasma protein biomarkers and childhood intellectual test performance. We applied proteomics to quantify proteins in plasma samples of 249 rural Nepalese children, 6–8 years of age who, 1 year later at 7–9 years of age, were administered the Universal Nonverbal Intelligence Test (UNIT). Among 751 plasma proteins quantified, 22 were associated with UNIT scores, passing a false discovery rate threshold of 5.0% (q < 0.05). UNIT scores were higher by 2.3–9.2 points for every 50% increase in relative abundance of two insulin-like growth factor binding proteins (IGFBPs), six subclasses of apolipoprotein (Apo) and transthyretin, and lower by 4.0–15.3 points for each 50% increase in relative abundance of 13 proteins predominantly involved in inflammation. Among them, IGFBP-acid labile subunit, orosomucoid 1 (ORM1), Apo C-I, and pyruvate kinase isoenzymes M1/M2 jointly explained 37% of the variance in UNIT scores. After additional adjustment for height-for-age Z-score and household socio-economic status as indicators of long-term nutritional and social stress, associations with 6 proteins involved in inflammation, including ORM1, α-1-antichymotrypsin, reticulocalbin 1, and 3 components of the complement cascade, remained significant (q < 0.05). Using untargeted proteomics, stable, constitutive facets of subclinical inflammation were associated with lower developmental test performance in this rural South Asian child population. Plasma proteomics may offer opportunities to identify functional, antecedent biomarkers of child cognitive development.

## Highlights

• Quantitative proteomics was applied to identify plasma proteins associated with child cognition.
• Nutritional status sensitive proteins were associated with increased intelligence test scores.
• Inflammatory proteins were associated with decreased test scores, independent of risk factors.
• A protein regulating glycolysis contributed to the variability in the test scores.
• Plasma proteomics can provide candidate proteins that reflect biological associations with child cognition.

## 1 Introduction

As child survival improves (The United Nations, 2015), attention is turning to assessing and alleviating coexisting burdens of stunted growth and suboptimal cognition among children in low and middle income countries (Black et al., 2013, Grantham-McGregor et al., 2007). Both conditions can be a consequence of undernutrition (Black et al., 2013, Walker et al., 2011) and share complexity and incomplete knowledge of their etiologies, challenging effective prevention (Gale, 2005). Yet, while the Sustainable Development Goals agenda for the year 2030 remains focused on reducing childhood growth faltering (The United Nations, 2014), similar multi-sectoral efforts to quantify the burden and develop interventions to prevent impaired cognition are lagging.

Difficulty and costs in deploying standardized tests across cultures represent clear obstacles to advancing the child development agenda (Prado et al., 2010). In addition, however, there remains population data on the complex biosocial interactions and risk factors of impaired cognition that could assist in identifying groups at risk (Turkheimer et al., 2003, Walker et al., 2007). Chronic exposure to protein-energy and micronutrient deficiencies (i.e., iron and iodine), infectious agents, environmental hazards, social stresses, and lack of stimulation and learning opportunities, all deeply rooted in poverty, have been identified as risk factors (Walker et al., 2011), but leaving the question how these insults disturb long-term cumulative biological processes of brain development unanswered.

During the past decade, emerging “-omics” studies have contributed to exploring biological pathways and discovering molecular footprints associated with neurodevelopmental disorders (Antonarakis et al., 2004, Bahado-Singh et al., 2013, Corbett et al., 2007, Woods et al., 2015). The high-throughput technologies are powerful in that they do not require prior knowledge, thereby enabling unprecedented discovery and allow analysis of hundreds of biological molecules with a single assay, providing comprehensive insights into the multifactorial nature of cognitive deficits. These advances, however, have yet to be applied to improve our understanding of common biological pathways that underlie suboptimal development of cognitive function of children in the developing world, where the most serious loss of developmental potential occurs (Grantham-McGregor et al., 2007).

The plasma proteome which is currently being explored for its potential to advance assessment of nutritional and health status of children (Cole et al., 2013, Lee et al., 2015, West et al., 2015) may provide a novel opportunity to identify biomarkers of molecular networks of homeostatic systems or other environmental exposures that affect cognitive function of children. The plasma protein milieu exhibits relative constancy within individuals over time and reflects a systemic “sum” of tissue status in the body (Anderson and Anderson, 2002). Neurotrophic and neuroendocrine factors that play key roles in neuronal cell signaling, synaptic plasticity and neurogenesis are present in plasma (Iughetti et al., 2011, O’Dorisio et al., 2002). In addition, the plasma proteome contains proteins whose expression are influenced by genetics, age, gender and nutritional and health status that are known to be underlying contributing factors of brain function (Dauncey, 2009). Plasma proteins influenced by some of these risk factors may not directly affect brain function, but can still indicate cognitive function as a systemic response to conditions that affect both protein abundance in plasma and developmental processes in the central nervous system (CNS).

In this study, we hypothesized that proteins that directly mediate or indirectly reflect cognitive function can be detected and quantified in plasma, and thus provide a repertoire of biomarkers that could provide insight into risk factors of poor cognition within or between populations. In southern Nepal, as part of a follow-up study in the offspring of women who participated in a prenatal micronutrient supplementation trial, we collected plasma samples at 6–8 years of age (Schulze et al., 2014, Stewart et al., 2009b). A year later, intellectual and motor functioning were assessed by standardized tests in a subgroup of the children at 7–9 years of age (Christian et al., 2010) at the time when the brain for higher-cognitive functions continues to mature (Grantham-McGregor et al., 2007). Using existing plasma samples and applying quantitative proteomics, we sought to identify plasma proteins that co-varied with cognition a year later, reflected by child performance on a standardized test of general intelligence.

## 2 Methods

### 2.1 Study design, population, and subjects

Children assessed in this study were a subset of a cohort of 4130 children born to 4926 mothers in the southern plains district of Sarlahi, Nepal who participated in a randomized controlled trial of antenatal micronutrient supplementation from 1999 to 2001 (ClinicalTrials.gov:NCT0011527) (Christian et al., 2003a, Christian et al., 2003b). Of these, 3673 children subsequently (2001–2004) participated in a second trial that evaluated growth effects of daily iron and/or zinc supplementation from 6 to 36 months of age, (Tielsch et al., 2006) forming part of their history of nutritional exposure. In 2006–2008, 3524 children in this cohort were re-assessed at 6–8 years of age for nutritional and health status, that included blood sample collection (Stewart et al., 2009a, Stewart et al., 2009b). Children with available plasma samples meeting inclusion criteria (n = 2130), were stratified by the five antenatal supplementation groups, each from which 200 children randomly selected for in-depth biochemical biomarker assessment (total n = 1000) (Schulze et al., 2014). From these, plasma samples of 100 (50%) children from each of 5 maternal micronutrient supplementation groups (total n = 500) were randomly selected for mass spectrometric proteomics analysis (Cole et al., 2013).

**Fig. 1:**
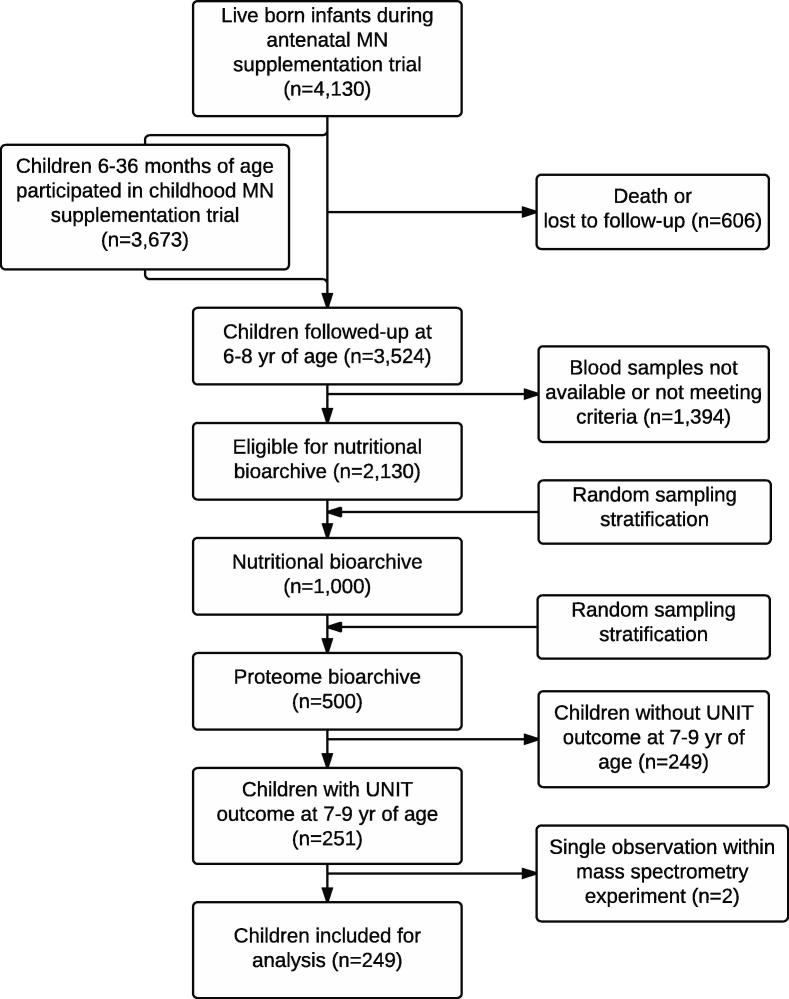
Consort diagram of study participants. Abbreviations: MN, micronutrient; UNIT, Universal Nonverbal Intelligence Test.

One year after the 2006-8 assessment, ∼1900 of the 3524 children were followed for neurocognitive and motor function testing, at 7–9 years of age (Christian et al., 2010). Thus, the present analysis was carried out on a subset of 251 children from the original trial whose records have both plasma proteomics data at 6–8 years of age and intelligence test data at 7–9 years (Fig. 1). Two children whose plasma samples appeared to be as a single sample within a mass spectrometry experiment were further excluded, yielding 249 children for analysis. Children in the present study (n = 249) were comparable based on numerous characteristics to other children in the proteomics study who were not assessed for cognitive function (also n = 249), except for a 0.6 kg heavier weight (*P* = 0.022) (data not shown). Participation in all studies required parental informed consent and all protocols were reviewed and approved by institutional review boards in Kathmandu, Nepal and Johns Hopkins Bloomberg School of Public Health, Baltimore, MD.

### 2.2 Assessment of general intelligence

Children’s intellectual functioning was assessed using the Universal Nonverbal Intelligence Test (UNIT). The UNIT was designed to provide a measure of general intelligence in a completely nonverbal format for children who would be unfairly assessed with a language-loaded ability test (Bracken et al., 1998). It measures mainly memory and reasoning abilities consisting of six subtests: symbolic, spatial, and object memory, analogic reasoning, cube design, and mazes. Details about the administration of the UNIT and the standardization of raw scores have been reported elsewhere (Christian et al., 2010). The analogic reasoning subtest was removed because it was not culturally appropriate to Nepalese children. Because this test has not been standardized in Nepal, factor structure was assessed using exploratory and confirmatory factor analyses. Total scores of the raw scores of five subtests were generated and were converted to *T*-scores (mean 50 and standard deviation 10) based on child’s age.

### 2.3 Child nutritional and health assessments and household socio-economic interview

Nutritional, health, demographic and socio-economic status (SES) assessments during the follow-up surveys have been described elsewhere (Stewart et al., 2009a, Stewart et al., 2009b). Briefly, during home visits, trained staffs asked heads of household about children’s years of schooling and household SES information (caste, religion, household asset ownership, parental education, and parental literacy). Anthropometrists measured children’s height and weight by standard procedures and collected 7-day histories of morbidity symptoms and dietary intake (Stewart et al., 2009a). Plasma concentrations of retinol, 25(OH)D, cobalamin, folic acid, transferrin receptor, ferritin, and thyroglobulin were measured to assess child vitamin A, D, and B12, folate, iron, and iodine status. For iron status, transferrin receptor to ferritin ratio (TfR:ferr) was used (Malope et al., 2001). High sensitive C-reactive protein (CRP) and alpha-1-acid glycoprotein (AGP), two widely used inflammation makers for population studies in low resource settings (Raiten et al., 2015), were measured by a benchtop clinical chemistry analyzer (Immulite 1000; Siemens Diagnostics) and a radial immunodiffusion assay (Kent Laboratories), respectively, using the same plasma specimens collected for proteomics analysis. Detailed information on biochemical measurements was published elsewhere (Schulze et al., 2014). Weight and height data were transformed to Z-scores based on the WHO reference growth charts for children aged 5–19 years using AnthroPlus software (de Onis et al., 2007).

### 2.4 Preparation of plasma samples and proteomics analysis

During the follow-up study in 2006-8, phlebotomists collected early morning fasting samples of venous blood (10 ml in sodium heparin-containing tubes without preservatives or antioxidants). The biospecimens were centrifuged for plasma extraction. Plasma was equally aliquoted into 4 tubes, frozen under liquid nitrogen, shipped to the Johns Hopkins University in Baltimore, MD, USA and stored at −80 °C.

Proteomics analyses procedures have been previously described (Cole et al., 2013). Briefly, plasma samples of the selected 500 children were immune-depleted to remove 6 highly abundant plasma proteins (albumin, immunoglobulin A, immunoglobulin G, transferrin, haptoglobin, and anti-trypsin) to enhance the detection of low abundant plasma proteins using a Human-6 Multiple Affinity Removal 200 System (MARS) LC column (Agilent Technologies). Immune-depleted samples were digested overnight by trypsin. Seven randomly selected samples from 500 children and one reference sample were randomly labeled with 8 isobaric tags for relative and absolute quantitation (iTRAQ) reagent (AB Sciex). All samples were mixed and fractionated into 24 fractions by strong cation exchange (SCX) chromatography. iTRAQ-labeled peptides were loaded to a reverse phase nanobore column and eluted peptides were sprayed into an LTQ Orbitrap Velos mass spectrometer (Thermo Scientific). MS/MS spectra were extracted using Thermo Scientific Xtract software and searched against the RefSeq 40 protein database using Mascot (Matrix Science) through Proteome Discoverer software (v1.3, Thermo Scientific). Peptides with high confidence (>95%) were filtered with a false Discovery Rate < 5%. Out of 72 iTRAQ experiments run for plasma samples of 500 children, 68 iTRAQ experiments for plasma samples of 249 children with intelligence test outcome were used for analysis.

### 2.5 Selection of potential risk factors

Because child sex, age, and prenatal and childhood iron-folic acid supplementation can be potentially associated with the outcome based on previous studies (Christian et al., 2010, Murray-Kolb et al., 2012), we considered the child characteristics as fixed covariates and adjusted for them as initial child characteristics in all models. In addition, we identified potential biological and psychosocial risk factors based on the literature and data analysis using uni- and multi-variate regression models (Supplementary Table 1); viz., child iron and iodine status, long-term nutritional status indexed by height-for-age Z-score (HAZ), body mass index Z-score, school attendance, food intake in the previous week, morbidity history in the past week, birth weight, ethnicity, caste, HOME inventory score, wealth index, and maternal literacy and education level (Christian et al., 2010, Christian et al., 2011, Christian et al., 2014, Patel et al., 2013, Walker et al., 2011). A household wealth index was created based on the first principal component of the polychoric correlation of multiple indicators of household assets (materials of ground, floor, and roof of house, bicycle, radio, television, electricity, cattle, goat, and land). We selected covariates independently associated with the outcome from the multivariate model. Among the selected variables, ethnicity (Pahadi originating from the hills and Madheshi originating from the plains), household wealth index, maternal education, and child school attendance were highly correlated with each other. We considered these as household socioeconomic and cultural risk factor variables and adjusted for them in fully adjusted models.

### 2.6 Adjusting UNIT *T*-score for risk factors

The mean (standard deviation) score of UNIT was 50.4 (10.6), ranging from 29.3 to 78.5. Because common underlying factors (i.e., household SES) can confound associations between plasma proteins and intellectual outcome or some plasma proteins may mediate the effects of underlying factors (i.e., nutritional status) on the outcome, we controlled for risk factor(s) to identify proteins associated with the identified risk factors for generating future hypotheses. We generated adjusted UNIT scores for different sets of the risk factors by taking residuals from regression models with UNIT *T*-score as a dependent variable and each or all risk factors as independent variables in addition to the fixed child characteristics.

### 2.7 Statistical analyses

The process of estimating relative abundance of proteins measured by multiple iTRAQ experiments has been described elsewhere (Herbrich et al., 2013). We employed linear mixed effects models (LME) to combine the proteomic data from different experiments and to estimate the association of protein relative abundances with the UNIT score outcome. We fit a univariate random intercept model with intelligence score as a dependent variable, each protein as a fixed effect, and an iTRAQ experiment as a random effect. Parameters were estimated using restricted maximum likelihood estimation (Harville, 1977). We transformed beta-coefficients (i.e., the fixed effect slope) to be interpreted as the estimated change in the adjusted UNIT score associated with a 50% increase (1.5 fold-change) in relative abundance of a protein. A p-value was calculated by testing the null hypothesis of no association between a protein and the UNIT score outcome. Multiple hypothesis testing was corrected by controlling the false discovery rate (FDR) (Storey, 2002). Proteins passing a FDR threshold of 5% (q < 0.05) were considered significantly associated with the outcome. The coefficient of determination (R^2^) was estimated by calculating the correlation between the intelligence test score and each respective best linear unbiased prediction from LME analysis (Robinson, 1991). As number of plasma samples loaded per iTRAQ experiment varied (ranged from 2 to 6), sensitivity analyses was performed by restricting iTRAQ experiments with >2 plasma samples to examine whether sample size per experiment affects association between protein and the outcome.

Because multiple proteins participating in the same biological processes can be highly correlated, we built a multiple-protein regression model to estimate the variability in the UNIT scores explained by a small subset of plasma proteins. Candidate proteins for the model were selected based on their marginal associations with the outcome (q < 0.05). The model started with a protein, which was most strongly associated with the outcome. Additional proteins, the next strongly associated with the outcome, were allowed to enter the model as covariates based on a forward stepwise procedure with a criterion that all included proteins should be significantly associated with the outcome (*P* < 0.05).

General information of proteins was derived from the National Center for Biotechnology Information (NCBI) Protein Database, UniProt Knowledgebase (Geer et al., 2010; The UniProt Consortium, 2014), and in-depth literature review. The dataset of UNIT score, covariates, and relative abundance of a subset of proteins is available in Supplementary Table 2. All analyses were carried out using open-source software implemented in the statistical environment R (R Development Core Team, 2004).

## 3 Results

### 3.1 Characteristics of study population

Characteristics of 249 children at their ages of blood collection (7.5±0.4 yr) and cognitive assessment a year later (8.4±0.7 yr) are summarized in Table 1, by which time about 67% and 80% of children had been to school. Although children had gained some weight and height, their nutritional status remained low and stable over the year with 35% stunted, 43% underweight, and 16% with a low body mass index (BMI) at both visits based on WHO criteria. Plasma micronutrient concentrations at the time of 1^st^ visit are summarized in Table 1. Deficiencies in vitamin A (retinol < 0.7 μmol/L), vitamin D (25(OH)D < 50 nmol/L), folate (folic acid < 13.6 nmol/L), vitamin B12 (cobalamin < 150 pmol/L), and iron (transferrin receptor to ferritin ratio > 500 μg/μg) were present in 8.4, 18.5, 6.4, 20.1, and 11.6% of children using conventional cut-offs (Schulze et al. 2014). Weekly dietary intakes were comparable at each visit, reflecting a rice-based diet supplemented with milk or curd, some vegetables and less frequent meat and egg intakes. Twenty-nine percent of children had an elevated plasma AGP, suggestive of prolonged inflammation, while 6% had a raised CRP, possibly reflecting more acute infection, and a rate that was comparable in magnitude those of lower respiratory infection (2%) and diarrhea (4%) in the past week.

### 3.2 Plasma proteins associated with the UNIT score

We analyzed a total of 751 proteins identified and quantified in more than any 50 plasma samples of children. Among them, twenty-two proteins (about 3% of total number analyzed) were associated with initial child characteristics (age, sex, and, prenatal and childhood micronutrient supplementation)-adjusted UNIT scores, passing a discovery threshold of 5% (q < 0.05) (Table 2). Nine proteins were positively associated with the outcome: insulin-like growth factor (IGF) binding protein acid labile subunit (IGFALS), IGF-binding protein 3 (IGFBP3), transthyretin (TTR), and 6 sub-classes apolipoproteins (APOA1, A2, C3, C1, D, and M). These proteins are known to transport or regulate insulin-like growth factors, thyroid hormone, and lipids/cholesterol in the circulatory system. UNIT scores increased by 2.3∼9.2 with a 50% increase in relative abundance of each protein, and the relative abundance of each protein accounted for 17.6–24.8% of the variance in the adjusted UNIT scores. Thirteen proteins were negatively associated with UNIT scores including orosomucoid 1 (ORM1 or AGP1), components of complement system (C2, C5, C9, and complement factor I), α-1-antichymotrypsin (SERPINA3 or ACT), leucine-rich α-2-glycoprotein 1 (LRG1), and lipopolysaccharide-binding protein (LBP). Most of these are acute phase proteins and others are intracellular proteins with various biological functions including glycolysis and the regulation of circadian rhythm. UNIT scores decreased by 4.0–15.3 per a 50% increase in relative abundance of each protein, explaining 18.5∼45.0% of the variance in the adjusted UNIT scores.

### 3.3 Proteins explained the variability in the UNIT score

A multiple-protein regression model showed that four proteins, including IGFALS, ORM1, APOC1, and pyruvate kinase isozymes M1/M2 (PKM) jointly explained 37% of the variance in the initial child characteristics-adjusted UNIT scores (R^2^) (basic model; Model A in Table 3). Additional adjustment for child iron status reflected by log transformed transferrin receptor to ferritin ratio did not materially change the associations with the 4 proteins and R^2^ (Model B in Table 3). However, controlling for the initial child characteristics and child HAZ attenuated about 50% of the observed association with IGFALS and reduced R^2^ to 30% (Model C in Table 3); controlling for the initial child characteristics plus household SES characteristics considerably attenuated the observed positive associations with IGFALS and APOC1, and reduced R^2^ to 13% (Model D in Table 3). When UNIT scores were adjusted for the initial child characteristics, HAZ, and household SES, negative associations with ORM1 and PKM remained significant, accounting for 11% of variance in the adjusted outcome (Model E in Table 3).

### 3.4 Plasma proteins associated with the UNIT score after adjusting for risk factors

Because adjusting for iron status did not substantially change R^2^, we fit a single-protein model again with UNIT scores adjusted for the initial child characteristics, child HAZ, and household SES characteristics. Out of 751 proteins tested, six proteins—ORM1, C9, CFI, ACT, reticulocalbin 1 (RCN1), and complement factor H-related 5 (CFHR5)—were negatively associated with the adjusted UNIT scores which decreased by 3.7–7.4 per a 50% increase in relative abundance of each protein (q < 0.05) (Table 4). These proteins are all known to be involved in inflammation. Other inflammatory proteins that did not pass the false discovery rate threshold of 5% included LRG1 (q = 0.0697), ORM2 or AGP2 (q = 0.0907), LBP (q = 0.0907), and CRP (q = 0.2911).

### 3.5 Validation of the proteomics findings with targeted inflammation markers

**Fig. 2:**
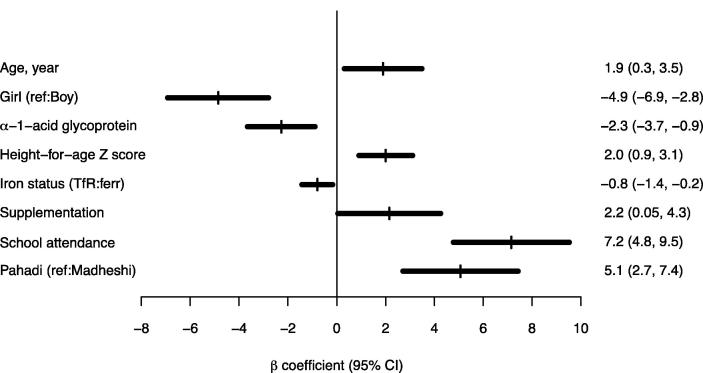
Associations between intelligence test score and alpha-1-acid glycoprotein (AGP) and other risk factors in a multivariate model in school-aged children in rural Nepal (n = 247). Abbreviation: Transferrin receptor to ferritin ratio, TfR:ferr. X-axis denotes expected difference (or change) in UNIT score adjusting for other covariates in the multivariate model. For AGP and iron status, estimated change in UNIT score was associated with a 50% increase in plasma AGP (g/L) and TfR:ferr (μg/μg) concentration, respectively. A high TfR:ferr value represents poor iron status. Supplementation represents children whose mothers received antenatal micronutrient supplements during their pregnancy that included iron and folic acid (vs. supplements that did not) during the original randomized clinical trial (Christian et al., 2010). Pahadi and Madheshi represent two major ethnic groups. Childhood iron-folic acid supplementation variable was included as a fixed covariate in the multivariate model [β (95% CI) = −1.4 (−3.5, 0.7), *P* = 0.1872] (not illustrated). Maternal education and household wealth index variables were not significantly associated with the outcome (*P* > 0.05) and dropped from the model.

In order to validate the observation that lower UNIT scores were associated with increased relative abundance of inflammatory proteins based on proteomics data, we examined associations between UNIT scores and two conventional inflammation markers, AGP and CRP, measured independently by conventional immunoassays. Children’s UNIT scores decreased by a mean of 2.3 (95% CI = 0.9, 3.7; *P* = 0.0015) per a 50% increase in plasma AGP concentration the year before adjusting for other risk factors (Fig. 2). The UNIT score was also less significantly negatively associated with CRP than AGP, evident by a 0.4 (95% CI = 0.1, 0.8; *P* = 0.0125) decrease per a 50% increase in CRP concentration, adjusting for the same covariates.

## 4 Discussion

Child development involves complex biological processes that may be compromised by chronic malnutrition and constant exposure to environmental hazards. Biomarkers may exist that broadly reflect such conditions that, if associated with developmental metabolic pathways, could help reveal biosocial causes and more informed ways to address poor cognition in impoverished settings. Using untargeted quantitative proteomics, this study among young school-aged children living in rural Nepal explored associations between plasma proteins and intellectual function, evaluated a year later by the UNIT, a measure of nonverbal general intelligence. Proteins that transport insulin-like growth factors, thyroid hormone, and lipids/cholesterol in circulation were associated with increased UNIT test scores, while proteins largely involved in inflammation were associated with decreased test scores that remained significant after adjusting for long-term nutritional and household socioeconomic status. Four proteins, representing potentially different biological pathways, jointly explained 37% of the variance in adjusted test scores of intellectual function. Collectively, these findings suggest that aspects of child cognitive development may be, in part, related to distinct but potentially interrelated biological pathways including nutritional and immunologic regulation.

### 4.1 Associations between inflammatory proteins and UNIT score

Intellectual test performance was negatively associated with several established proteins known to be involved in systemic inflammation, including ORM1, complement components, ACT, and LBP, lending credibility to similarly negative associations observed with less well known reactants. Although biological functions of LRG1, RCN1, leucine-rich repeat-containing protein 50, and ecotropic viral integration site 5 protein remain elusive, our previous study revealed that they are part of a systemic response to inflammation (Lee et al., 2015). Negative associations of ORM1, ACT, RCN1, and complement components with the UNIT score, independent of chronic stressors known to be associated with poor development in poor societies, reflected by a childhood height-for-age and household socioeconomic status (Patel et al., 2013, Walker et al., 2011), implicate subclinical inflammation in repressing cognitive development.

There is convincing prospective epidemiological evidence that peripheral inflammation adversely affects cognition. In Bangladesh, pro-inflammatory cytokines at 6 months of age were associated with decreased motor scores at 24 months of age, independent of linear growth at 6 months, family income, and maternal education (Jiang et al., 2014). Among adult cohorts in developed countries, inverse associations have been noted between elevated concentrations of inflammation markers and cognitive ability, controlling for health and socio-economic status (Gimeno et al., 2008, Karlsson et al., 2010). These findings support a complex and sustained communication between the peripheral immune system and the CNS function.

It is plausible that cytokines, acting as upstream effectors of identified inflammatory proteins, may play key roles in mediating the effects of peripheral inflammation on the CNS (Wilson et al., 2002). Although not detected by mass spectrometry in this study possibly due to their low abundances in the circulation (Anderson and Anderson, 2002), pro-inflammatory cytokines such as IL-6 and TNF-alpha are known to induce a systemic acute phase response in the peripheral compartment (Gabay and Kushner, 1999). Circulating cytokines can also induce inflammation in the CNS believed to be responsible for disturbances in memory consolidation and synaptic plasticity, and poor cognition (Chen et al., 2008, Krabbe et al., 2005, Reichenberg et al., 2001) with or without crossing the blood-brain barrier (Quan and Banks, 2007). Other mechanisms may also exist that enable access of circulating acute phase proteins (e.g. AGP and ACT) to the CNS (Adam et al., 2001, Goodman and Vulpe, 1961), but an insufficient number of studies currently exist to establish these mechanisms.

Our proteomics findings suggest that some inflammatory proteins such as ORM1 (i.e., AGP1), ACT, and complement components (all q < 0.05 in Table 4) are more strongly associated with intelligence test performance than other well-known inflammatory proteins. For example, CRP, one of the most widely used inflammation biomarkers, was significantly associated with the outcome based on a conventional level of significance (*P* = 0.0241), but did not pass the false discovery rate threshold of 5% (q < 0.05) after multiple comparison correction (q = 0.2911). These findings were consistent with results of two targeted inflammation markers, CRP and AGP, that were quantified independently by conventional immunoassays (Results 3.5). Other studies have also reported varying degree of associations depending on inflammatory markers in older persons (Dik et al., 2005, Engelhart et al., 2004, Singh-Manoux et al., 2014). In this population characterized by chronic, subclinical inflammation, stable undernourishment reflected by anthropometric indicators, and a low prevalence of acute infections (Schulze et al., 2014), it is possible that mild but prolonged inflammation reflected by AGP or complement components constitutively present in circulation and moderately reactive during inflammation (Mackiewicz et al., 1993, Thurnham and McCabe, 2012) may disturb developmental cognitive processes of children. As these proteins have been rarely evaluated in relation to cognition, this study identifies additional inflammatory markers for investigations of suboptimal intellectual function in populations where chronic inflammation and undernutrition are common.

### 4.2 Associations between insulin-like growth factor binding proteins and UNIT score

Two IGF-binding proteins were positively associated with intellectual test performance a year later: IGFALS and IGFBP3, both known to form ternary complexes with IGF1 in circulation and modulate its bioavailability (Boisclair et al., 2001). IGF1 itself, however, was not associated with the outcome (q=0.236), disputing a posited role for peripheral IGF1 in cognitive development at this childhood age (Berger, 2001, Popken et al., 2005). In British school-aged children, serum IGF1 concentration was associated with a verbal component of intelligence quotient test scores (Gunnell et al., 2005). Discrepant results between studies may relate to differences in living standards, and nutritional and education status of children, as well as different domains of intellectual function being tested. As our work is also revealing IGFALS and IGFBP3 to be the most sensitive of identified plasma proteins associated with height-for-age (Lee et al*.* unpublished results, 2016), it is possible that the observed positive associations indirectly reflect other biological pathways, such as those involving growth hormone on cognitive development (Nyberg and Hallberg, 2013) or other strong but non-causal relationships between height and intelligence (Gale, 2005).

### 4.3 Associations between apolipoproteins and UNIT score

Plasma abundance of six apolipoproteins was positively associated with subsequent UNIT test scores, which in our larger sample of children (n = 500) showed strong positive associations with HDL (all *P* < 0.0001). Evidence is currently lacking in industrialized countries that peripheral apolipoproteins or cholesterol are associated with childhood cognitive function (Perry et al., 2009, Rask-Nissila et al., 2000). As cholesterol in the brain is virtually isolated from cholesterol in peripheral circulation (Bjorkhem and Meaney, 2004), the observed positive associations may represent confounding by household socioeconomic status that can be positively related to lipid profiles and cognitive performance of children. However, we cannot rule out roles for plasma cholesterol modulating the immune system without crossing the blood-brain barrier (Sena et al., 2012). There is also evidence that APOA1, which exerts anti-inflammatory functions in the CNS, is supplied from circulating APOA1 in the plasma (Vitali et al., 2014). Without studies in low-income countries where dietary quality is often low, it may be too early to rebut potential effects of peripheral apolipoprotein profiles on cognitive function.

### 4.4 Association between pyruvate kinase isoenzymes M1/M2 and UNIT score

PKM was selected as a negative covariate in the multiple protein regression model with IGFALS, ORM1, and APOC1 suggesting possible antagonism on cognitive processes. It is a rate-limiting enzyme in glycolysis mediating cellular energy production (Muirhead, 1990). It is known to act as a metabolic (glucose) sensor that regulates cell growth, size, and apoptosis (Spoden et al. 2009). In addition, studies suggest that it plays a critical role in sustaining immunologic response of peripheral lymphocytes and activating the innate immunity against pathogenic infections (Burge et al., 1976, Marjanovic et al., 1991). Although studies have been lacking associating PKM to cognition, we postulate that the negative association observed in this study indicates increased metabolic stress or requirement for cellular growth and immunologic response (Gupta and Bamezai, 2010) that can potentially compromise developmental processes in the CNS. Collectively, the results from the multiple protein regression model have revealed diverse biological pathways, including systemic inflammation, regulation of insulin-like growth factors, lipid transport/metabolism, and cellular energy metabolism that may contribute to a complex biology of childhood cognitive development.

### 4.5 Strengths and limitations

To our knowledge, this is the first study that has attempted to quantitatively and temporally associate plasma protein abundance to general intelligence test score, providing a systems perspective to assessing child cognitive function, in a typically undernourished South Asian setting. The untargeted proteomics approach enabled us to detect a novel suite of plasma protein biomarkers whose shared biological pathways strengthen the plausibility of the findings. The year lag between blood sampling and cognitive function testing (i) enabled us to rule out associations due to co-existing, episodic health conditions that could occur in cross-sectional assessments of protein abundance and test performance and (ii) suggests that measures of protein abundance provide a stable and refined index of protein status over time, as was observed with anthropometric status in this population. Although this study included a small fraction of a population cohort (Christian et al., 2010, Stewart et al., 2009a), studied children were typical in their nutrition and health profiles to other child populations in rural Nepal, minimizing concern about bias. Further, rich demographic and nutritional data extending from prenatal to postnatal life allowed us to account in the analysis for multiple risk factors of child development.

Among the study’s limitations, many identified proteins are viewed as ‘classic’ plasma proteins that may not be directly mediating cognitive function. Neuropeptides or neurotrophins, such as brain-derived neurotrophic factor, that are known to modulate neural and cognitive function (Cohen-Cory et al., 2010, Gomez-Pinilla, 2008) were not detected by mass spectrometry. These proteins/peptides are generally present at low plasma concentrations (pg/ml) (Iughetti et al., 2011, O’Dorisio et al., 2002) and, thus, were probably undetectable under present study conditions. Finally, while the range of changes in UNIT scores per increase in protein abundance cannot be directly compared to other studies because proteins were not quantified in an absolute scale, relative abundance estimation has been shown to be valid in revealing the direction and strength of protein associations with other outcomes, such as aspects of nutritional status (Schulze et al., 2016, West et al., 2015).

## 5 Conclusions

This exploratory study revealed that plasma proteomics can identify candidate proteins associated with intellectual test performance in a typical, rural child population in South Asia. As the importance of assessing subclinical inflammation is under-recognized in the integrative child development agenda, additional studies are needed to confirm associations between prolonged subclinical inflammation and childhood cognitive function which can stimulate investigation into the causes and prevention of chronic inflammation early in life. Plasma proteins such as those identified in this study could serve dual roles as functional indicators at the intersection of both environmental stress and child development.

## Conflict-of-interest

The authors declare no conflict of interest.

## Authorship

K.P.W., R.N.C., K.J.S., J.D.Y, J.D.G., and P.C. designed the proteomics research and P.C. designed the Nutrition and the Cognition study. R.N.C. and K.J.S. supervised laboratory work for the proteomics study. P.C. has a full access to data of cognitive outcomes. L.S.F.W. managed dataset. S.E.L. analyzed data. K.P.W., L.S.F.W., and P.C. conducted the original field study. S.E.L. drafted the manuscript. S.E.L. had primary responsibility for final content. All authors read and approved the final manuscript.

## Figures and Tables

**Table 1:** Demographic, nutritional, health, and dietary characteristics of Nepalese children included in analysis at the time of blood sampling and cognitive assessment (n = 249)

Characteristics	Values at blood sampling (2006-8)^a^	Values at cognitive assessment (2007-9)^a^
Age, years	7.5 (0.4)	8.4 (0.7)
Ever sent to school, n (%)	167 (67.1)	198 (79.5)
Male, n (%)	122 (49.0)	122 (49.0)

*Anthropometric measurements*		
Weight, kg	18.6 (2.9)	20.6 (3.2)
Height, cm	114.5 (5.9)	119.4 (6.3)
BMI, kg/m^2^	14.1 (1.2)	14.4 (1.4)
Stunting (HAZ^b^ < −2), n (%)	88 (35.3)	91 (36.5)
Underweight (WAZ^b^ < −2), n (%)	108 (43.4)	107 (43.0)
Low BMI (BMIZ^b^ < −2), n (%)	41 (16.5)	40 (16.1)

*Plasma micronutrient status indicators*^c^		
Retinol, μmol/L	1.05 (0.26)	–
25(OH)D, nmol/L	66.2 (18.1)	–
Folic acid, nmol/L	23.8 (19.2, 30.4)^d^	–
Cobalamin, pmol/L	236.3 (112.1)^d^	–
TfR:ferr, μg/μg	203 (134, 317)^d^	–

*Diet in the past 7 days (⩾3 times), n (%)*		
Rice, corn, and wheat	248 (99.6)	249 (100.0)
Milk and curd	119 (47.8)	111 (44.6)
Egg	6 (2.4)	5 (2.0)
Fish, chicken, and other meat	17 (6.8)	29 (11.6)
Yellow vegetables and fruit^e^	39 (15.7)	42 (16.9)
Dark green leafy vegetables	75 (30.1)	93 (37.3)

*Plasma inflammation markers*^c^*, n(%)*		
AGP > 1.0 g/L	71 (28.5)	–
CRP > 5 mg/L	14 (5.6)^d^	–

*Morbidity in the past 7 day, n (%)*		
Lower respiratory infection^f^	5 (2.0)	6 (2.4)^d^
Diarrhea^g^	11 (4.4)	11 (4.4)

Abbreviations: BMI, body mass index; WAZ, weight-for-age Z-score; HAZ, height-for-age Z-score; BMIZ, body mass index Z-score; TfR:ferr, transferrin receptor to ferritin ratio; AGP, α-1-acid glycoprotein; CRP, C-reactive protein.

a Data are given as mean (standard deviation) or median (interquartile ranges) if distributions were skewed unless otherwise indicated.

b Z-score was defined by the WHO Growth reference 5–19 years.

c Blood samples were not collected at the time of cognitive assessment.

d Data are missing for folic acid (n=1), cobalamin (n=1), ferritin (n=2), and lower respiratory infection (n=2), and one outlier value for CRP was excluded.

e Yellow vegetables and fruit group includes mango, papaya, jackfruit, and pumpkin.

f Lower respiratory infection was defined by a history of productive cough or rapid breathing and fever in the past week.

g Diarrhea was defined by watery stools or dysentery in the past week.

**Table 2:** Plasma proteins positively and negatively associated with intelligence test score in school-aged children in rural Nepal (q < 0.05)

Molecular/biological function	Protein name (gene symbol)^a^	n^b^	β (95% CI)^c^	R^2^^d^	Accession^e^
*Positively associated proteins*					
Transport/regulate insulin-like growth factors (Baxter, 1990)	IGF-binding protein acid labile subunit (IGFALS)	249	7.1 (4.3, 9.9)	24.8	4826772
	IGF-binding protein 3 (IGFBP3)	249	4.4 (1.9, 6.9)	18.6	62243068
Transport thyroxine and retinol (Kanai et al., 1968, Woeber and Ingbar, 1968)	Transthyretin (TTR)	249	9.2 (4.8, 13.7)	20.8	4507725
Transport lipids/cholesterol (Alaupovic, 1982)	Apolipoprotein A-I (APOA1)	249	7.4 (3.7, 11.1)	20.3	4557321
	Apolipoprotein A-II (APOA2)	249	6.6 (3.1, 10.0)	19.6	4502149
	Apolipoprotein C-III (APOC3)	249	3.6 (1.5, 5.7)	18.3	4557323
	Apolipoprotein C-I (APOC1)	249	2.3 (0.9, 3.7)	17.9	4502157
	Apolipoprotein M (APOM)	249	5.8 (2.1, 9.5)	17.6	22091452
	Apolipoprotein D (APOD)	249	5.2 (1.9, 8.6)	17.6	4502163

*Negatively associated proteins*					
Acute phase response (Gabay and Kushner, 1999)	Orosomucoid 1 (ORM1) or α-1-acid glycoprotein 1 (AGP1)	249	−5.3 (−7.5, −3.1)	23.5	167857790
Complement cascade (Janeway et al., 2001)	Complement component 9 (C9)	249	−7.6 (−11.0, −4.3)	22.4	4502511
	Complement factor I (CFI)	249	−9.9 (−14.3, −5.4)	22.0	119392081
	Complement component 2 (C2)	211	−15.3 (−22.7, −7.9)	23.0	14550407
	Complement component 5 (C5)	249	−8.4 (−13.2, −3.7)	18.7	38016947
Acute phase response (protease) (Gabay and Kushner, 1999)	α-1-antichymotrypsin (SERPINA3 or ACT)	249	−9.8 (−14.2, −5.4)	22.2	50659080
Angiogenesis (Wang et al., 2013)	Leucine-rich α-2-glycoprotein 1 (LRG1)	249	−5.0 (−7.6, −2.5)	19.9	16418467
ER calcium-binding protein (Ozawa and Muramatsu, 1993)	Reticulocalbin 1 (RCN1)	59	−6.2 (−9.6, −2.9)	43.2	4506455
Regulate genome stability, cell survival, the circardian clock (Engelen et al., 2013, Sakai et al., 2004)	Protein timeless homolog (TIMELESS)	62	−13.4 (−21.1, −5.8)	31.6	222136585
Acute phase response (Gabay and Kushner, 1999)	Lipopolysaccharide-binding protein (LBP)	249	−4.0 (−6.3, −1.7)	18.5	31652249
Glycolysis (Marjanovic et al., 1990)	Pyruvate kinase isozymes M1/M2 (PKM)	239	−6.1 (−9.8, −2.3)	20.7	33286418
Regulate of microtubule-based cilia function (van Rooijen et al., 2008)	Leucine-rich repeat-containing protein 50 (DNAAF1)	107	−4.4 (−7.2, −1.7)	45.0	157674358
Modulate cell cycle, cytokinesis, and cellular membrane traffic (Lim and Tang, 2013)	Ecotropic viral integration site 5 protein (EVI5)	134	−4.5 (−7.2, −1.7)	27.8	68299759

Abbreviations: IGF, insulin-like growth factor; ER, endoplasmic reticulum.

a A list of proteins passing a false discovery rate threshold of 5% (q < 0.05) among a total of 751 plasma proteins (number of child plasma sample > 50) tested by linear mixed effect models. Proteins are ordered by direction of association and clustered by similar molecular/biological function.

b Number of child plasma samples in which a protein was detected and quantified by iTRAQ tandem mass spectrometry

c Estimated change in child characteristic (age, sex, and, prenatal and child micronutrient supplementation)-adjusted UNIT score per a 50% increase in relative abundance of each protein.

d Percentage of variance in child characteristics-adjusted UNIT scores explained by protein.

e GenInfo sequence number as assigned to all nucleotide and protein sequences by the National Center for Biotechnology Information at the National Library of Medicine.

**Table 3:** Association between selected plasma proteins and intelligence test score in multiple-protein regression model in school-aged children in rural Nepal (n = 237)^a^

Gene symbol	Model A^b^		Model B^c^		Model C^d^		Model D^e^		Model E^f^	
β (95% CI)^g^	*P*^h^	β (95% CI)^g^	*P*^h^	β (95% CI)^g^	*P*^h^	β (95% CI)^g^	*P*^h^	β (95% CI)^g^	*P*^h^
IGFALS	4.9 (2.0, 7.8)	0.0008	4.8 (2.0, 7.6)	0.0008	2.4 (−0.4, 5.2)	0.0965	1.5 (−1.1, 4.1)	0.2638	0.0 (−2.6, 2.5)	0.9786
ORM1	−3.5 (−5.8, −1.3)	0.0021	−4.0 (−6.2, −1.9)	0.0003	−3.4 (−5.6, −1.2)	0.0021	−3.4 (−5.4, −1.3)	0.0011	−3.5 (−5.4, −1.5)	0.0006
APOC1	1.4 (0.0, 2.8)	0.0478	1.2 (−0.2, 2.5)	0.0867	1.2 (−0.1, 2.6)	0.0725	0.6 (−0.6, 1.9)	0.3203	0.6 (−0.6, 1.8)	0.3360
PKM	−4.6 (−8.1, −1.1)	0.0094	−4.8 (−8.2, −1.4)	0.0057	−5.0 (−8.4, −1.6)	0.0042	−3.9 (−7.1, −0.7)	0.0168	−4.2 (−7.3, −1.1)	0.0072
R^2^^i^	0.37		0.38		0.30		0.13		0.11	

Abbreviation: IGFALS, insulin-like growth factor binding protein acid labile subunit; ORM1, orosomucoid 1; APOC1, apolipoprotein C-I; PKM, pyruvate kinase isozymes M1/M2.

a Analysis was restricted to children (n = 237) with completed data for UNIT scores, selected 4 proteins and all covariates included in Model A-E.

b Model A: UNIT scores were adjusted for initial child characteristics (child age, sex, and prenatal and child micronutrient supplementation) (basic model).

c Model B: UNIT scores were adjusted for initial child characteristics and iron status (log transformed transferrin receptor to ferritin ratio).

d Model C: UNIT scores were adjusted for initial child characteristics and child height-for-age Z-score (HAZ).

e Model D: UNIT scores were adjusted for initial child characteristics and household characteristics (wealth index, maternal education, ethnicity, and schooling).

f Model E: UNIT scores were adjusted for initial child characteristics, HAZ, and household characteristics (fully adjusted model)

g Estimated change in adjusted UNIT score per a 50% increase in relative abundance of single protein in a multiple-protein regression model

h P-value calculated by testing the null hypothesis of no association between single protein and adjusted UNIT score in the multiple-protein regression model

i Total variance in adjusted UNIT scores explained by proteins in each model.

**Table 4:** Plasma proteins associated with intelligence test score adjusted for initial child characteristics, height-for-age Z-score, and household characteristics in school-aged children in rural Nepal (q < 0.05)^a^

Molecular/biological function	Protein name (gene symbol)	n^b^	β (95% CI)^c^	R^2^^d^	Accession^e^
Acute phase response (Gabay and Kushner, 1999)	Orosomucoid 1 (ORM1) or α-1-acid glycoprotein 1 (AGP1)	249	−3.7 (−5.6, −1.9)	6.0	167857790
Complement cascade (Janeway et al., 2001)	Complement component 9 (C9)	249	−6.1 (−8.9, −3.3)	7.0	4502511
	Complement factor I (CFI)	249	−7.4 (−11.1, −3.7)	5.8	119392081
	Complement factor H-related 5 (CFHR5)	241	−4.7 (−7.0, −2.3)	6.7	13540563
Acute phase response (protease) (Gabay and Kushner, 1999)	α-1-antichymotrypsin (SERPINA3 or ACT)	249	−6.9 (−10.6, −3.2)	5.2	50659080
ER calcium-binding protein (Ozawa and Muramatsu, 1993)	Reticulocalbin 1 (RCN1)	59	−5.0 (−7.7, −2.3)	38.6	4506455

Abbreviations: ER, endoplasmic reticulum.

a A list of proteins passing a false discovery rate threshold of 5% (q < 0.05) among a total of 751 plasma proteins (number of child plasma sample > 50) tested by linear mixed effect models. Proteins are ordered by molecular/biological function.

b Number of child plasma samples in which a protein was detected and quantified by iTRAQ tandem mass spectrometry.

c Estimated change in initial child characteristic (age, sex, and previous micronutrient supplementation), child height-for-age Z-score (HAZ), and household characteristics (wealth index, maternal education, ethnicity, and child school attendance)-adjusted UNIT scores per a 50% increase in relative abundance of each protein.

d Percentage of variance in initial child characteristics, HAZ, and household characteristics adjusted-UNIT scores explained by each protein.

e GenInfo sequence number as assigned to all nucleotide and protein sequences by the National Center for Biotechnology Information at the National Library of Medicine.
