# Supplementary material for: General intelligence is associated with subclinical inflammation in Nepalese children: A population-based plasma proteomics study
Source: Brain Behav Immun. 2016 Aug;56:253–63. doi: 10.1016/j.bbi.2016.03.023 (PMC4929134; doi:10.1016/j.bbi.2016.03.023)
Supplement: Supplementary Table 1 [file mmc1.docx]

**Supplementary Table 1.** Associations between intelligence test score and potential risk factors of child development in school-aged children in rural Nepal

|  | Univariate  (n=249) | Multivariate^1^  (n=247) |
| --- | --- | --- |
| Variables | β (95% CI) | β (95% CI) |
| Age, year | 1.1 (-0.9, 3.1) | 1.8 (0.3, 3.4) |
| Girl (ref: Boy) | -5.5 (-8.1, -3.0) | -4.7 (-6.8, -2.6) |
| Prenatal IFA supplementation (ref: supplements not containing IFA)^2^ | 2.2 (-0.5, 4.9) | 1.7 (-0.5, 3.8) |
| Child IFA supplementation (ref: supplements not containing IFA)^3^ | -2.3 (-4.9, 0.3) | -1.3 (-3.4, 0.7) |
| Ever sent to school | 11.0 (8.5, 13.4) | 6.6 (4.1, 9.0) |
| HAZ | 3.3 (2.1, 4.6) | 2.1 (1.0, 3.2) |
| BAZ | 1.7 (0.2, 3.2) | - |
| Iron status^4,5^ | -2.4 (-4.3, -0.5) | -1.5 (-3.0, 0.0) |
| Iodine status^5,6^ | -1.2 (-2.9, 0.51) | - |
| WAZ at birth | 2.4 (1.1, 3.7) | - |
| Lower respiratory infection^5,7^ | -4.9 (-14.3, 4.5) | - |
| Diarrhea^8^ | -2.5 (-8.9, 3.9) | - |
| Ethnicity, Pahadi (ref: Madheshi) | 8.8 (6.2, 11.4) | 3.3 (0.7, 6.0) |
| Caste (ref: Non-Hindu) |  | - |
| Vaisha | 6.3 (3.2, 9.5) | - |
| Brahmin or Chhetri | 12.2 (8.2, 16.2) | - |
| Wealth index^9^ | -2.2 (-2.9, -1.5) | -0.7 (-1.4, 0.0) |
| HOME inventory score^5,10^ | 0.5 (0.3, 0.7) | - |
| Maternal education | 1.3 (0.9, 1.7) | 0.4 (0.0, 0.8) |
| Maternal literacy | 8.4 (5.5, 11.2) | - |
| R^2^ % | - | 43.4% |

Abbreviations: IFA, iron-folic acid; HAZ, height-for-age Z-score; BAZ, body mass index-for-age Z-score; WAZ, weight-for-age Z-score.

^1^Child age, sex, and prenatal/childhood micronutrient supplementation were fixed variables. Other variables were selected based on independent associations with UNIT score.

^2^Prenatal IFA indicates antenatal micronutrient supplements containing iron and folic acid.

^3^Child IFA indicates childhood micronutrient supplements containing iron and folic acid.

^4^Iron status was defined by log-transformed transferrin receptor to ferritin ratio (μg /μg). High values represent low iron status.

^5^Data are missing for iron status (n=2), iodine stats (n=2), lower respiratory infection (n=2), HOME inventory score (n=1).

^6^Iodine status was defined by log-transformed thyroglobulin (μg/L).

^7^Lower respiratory infection was defined by episode of productive cough or rapid breathing and fever in the past week.

^8^Diarrhea was defined by episode of watery stools or dysentery in the past week.

^9^High scores represent low assets.

^10^Environmental influences on child outcomes were indexed with the Middle Childhood Home Observation for the Measurement of the Environment (HOME) Inventory.
